# Growth, maturation and injuries in high-level youth football (soccer): A mini review

**DOI:** 10.3389/fspor.2022.975900

**Published:** 2022-11-01

**Authors:** Eirik Halvorsen Wik

**Affiliations:** ^1^Department of Exercise, Sport and Lifestyle Medicine, Faculty of Medicine and Health Sciences, Institute of Sport and Exercise Medicine, Stellenbosch University, Stellenbosch, South Africa; ^2^Division of Physiotherapy, Department of Health and Rehabilitation Sciences, Faculty of Medicine and Health Sciences, Stellenbosch University, Stellenbosch, South Africa

**Keywords:** soccer, sports medicine, growth and development, epidemiology, youth

## Abstract

Understanding the challenges football (soccer) players face during adolescence is fundamental to avoid disruptions in their development due to injury. This mini review will describe basic concepts of somatic growth and biological maturity, examine data from 53 prospective epidemiological studies on high-level youth football players and discuss how age, growth and maturity may affect the injury patterns observed. Based on the existing evidence, at least every third player sustains an injury during a football season. The thigh (median for studies of boys: 25%, median for girls: 21%), ankle (b: 18%, g: 30%), knee (b: 17%, g: 18%) and hip/groin (b: 14%, g: 10%) are the body parts injured most often, while muscle strains (b: 31%, g: 25%), sprains (b: 20%, g: 27%) and contusions (b: 17%, g: 16%) are the most common injury types. Injury trends are, however, not consistent throughout adolescence, and players' age, maturity status and position relative to peak height velocity (PHV) have shown to influence the number, type and location of injuries sustained. Despite a high volume of observational injury studies published on high-level youth players, girls (7 studies) and settings outside of Europe (included in 23% of studies) are underrepresented and should receive extra attention in the future. Based on the available epidemiological data, tailored injury reduction programmes can be considered in youth football, alongside application of general training principles such as progression, variation and individualization which may be especially important during vulnerable phases such as the adolescent growth spurt.

## Introduction

If you have been involved in youth football (soccer) in any capacity – that be as a player, coach, parent, physiotherapist or team coordinator – you probably have at least one story about “that player who grew 10 cm over the summer,” “seemingly fully-grown adults playing alongside children” or “the player who could have reached the top if it wasn't for that injury.” Stories like these make youth football both interesting and challenging, with some unique obstacles not seen at the senior level.

A general understanding of the changes adolescents experience when transitioning from children to adults [see Malina et al. (1)] is essential for anyone working in youth sports, and awareness around issues relating to injury risk may allow more talents to stay in their sport and develop to their full potential. Injuries keep players out of sessions and disrupt their development, which again may lead to them being dropped from a development programme (2). In some instances, they can have long-term health consequences (3). Although preventing all injuries is near impossible, it is in everyone's interest to limit the frequency and severity of injury. In this mini review, we will explore typical injury patterns in youth football and examine how growth and biological maturation may affect the chances of sustaining one.

## Understanding concepts of growth and maturity

Phrases like “the growth spurt,” “maturity timing” or “maturity status” can be confusing if used without a clear indication of what they refer to. In research, inconsistent terminology complicates aggregation of findings and in practice it may be a barrier for clear communication within a coaching team or to players and parents. The aim of this first section is therefore to define and clarify some key concepts. Although this review focuses on somatic (bodily) growth and biological maturation, it should be acknowledged that other aspects not covered, such as cognitive, behavioral and social development, or development of motor and psychological skills, also may affect the risk of sustaining injuries (4), which are considered both multifactorial, dynamic and complex (5–7).

### Somatic growth and the adolescent spurt

Growth can be defined as a change in the size of the whole body or a body part (1). A player's growth can therefore be assessed by measuring changes in physical dimensions (e.g., height, weight or leg length) over time. Growth in height follows a distinct non-linear pattern from birth to adulthood, with rapid changes observed right after birth, relatively steady growth throughout childhood, a new period of high acceleration during puberty, followed by a deceleration until adult height is reached (8).

The changes around puberty are especially interesting in the context of youth football, as the “adolescent growth spurt” takes off around the age of 8–10 years in girls and 10–12 years in boys (9, 10). The point of maximal acceleration (peak height velocity; PHV), where typical height velocities are around 7–9 cm/year (girls) and 8–10 cm/year (boys), occurs at a younger age in girls (around 11–13 years) compared to boys (around 13–15 years) (9, 10). There is, however, large variation in timing and magnitude between individuals, where age at PHV (timing) can range from 9 to 15 (girls) and 12 to 17 (boys) years, and maximal growth velocities (magnitude) can range from 5 to 10 (girls) and 5–12 (boys) cm/year (9, 10). Variation can also be seen between body parts in the same individual, where distal bones typically reach their peak velocity at a younger age compared to bones located higher up (1).

Adolescents also experience a period of accelerated weight gain: peak weight velocity (PWV). Maximal gains around 7–9 kg/year in girls and 9–11 kg/year in boys are common, around the ages of 12–14 years in girls (range: 11–15) and 13–15 years in boys (range: 13–16) (11). It is worth noting that girls, in general, gain proportionally more fat mass while boys add more lean mass (e.g., muscle and bone) (1). These relatively fast changes in height, weight and body composition at varying ages are important to consider, as they can result in large height and weight differences within age groups (12) and may impact both neuromotor coordination and injury risk negatively (13, 14).

### Making sense of biological maturity

The concept of growth can be conceptually easy to grasp; maturation on the other hand, is more complex and refers to the progress toward a mature (adult) state (1). In essence, this implies that a specific biological system has a certain end point (i.e., the mature state), and *maturation* is the journey to reach this endpoint. The end point depends on the system we are looking at; for example, the skeleton starts off as cartilage and matures to ossified bone (skeletal maturity), while sexual maturity is reached with full reproductive function (1, 15).

Adolescence is a phase associated with particularly large changes in different biological systems relating to the onset of puberty (16). The sequence of puberty often follows a typical pattern; however, there will be variation between girls and boys, and between individuals (16). The age at reaching certain maturational landmarks (e.g., PHV or the onset of menstrual cycles) is what we refer to as *maturity timing*. When comparing similar indicators, these are typically reached at a younger age in girls than in boys (1). The rate of change or time between maturational events (*maturity tempo*) also varies, meaning that some will be more advanced than others, even if their chronological age is the same. How far an individual has come at a given time point is what we mean by *maturity status*. In youth football, differences in maturity status are particularly relevant since players most often compete in chronological, not maturity-based, age groups.

## Injury patterns in high-level youth football

As mentioned in the introduction, one purpose of reducing the impact of injuries is to maximize developmental opportunities and performance. Understanding injury patterns is therefore fundamental, as we need to know which problems to focus on in order to best mitigate risk (17). The focus in this section will be on high-level adolescent players (e.g., elite, academy, professional club), including data from 53 prospective studies (per March 2022) which reported overall injury outcomes for minimum one season. Although methodological differences (e.g., injury definitions, recorders, classification systems) make comparisons and data aggregation difficult, and relatively few studies have been published on high-level female players, some trends are apparent.

### How common and severe are football injuries?

One approach to determining the extent of injuries is to count how many players sustain at least one injury over a season. In boys, this has ranged from 38 to 85% (18–25), with 0.4–2.2 injuries per player per season (19–42). Only one study reported seasonal injury proportion for girls (37%) (43), with another finding an average of 4.3 injuries per player per season (44). The latter also revealed that every second female player experienced an injury problem affecting performance, participation or pain during a given week (44). This suggests that injuries are indeed common in youth football, with a conservative estimate suggesting that at least every third high-level player will be injured once or more during a season.

**Figure 1:**
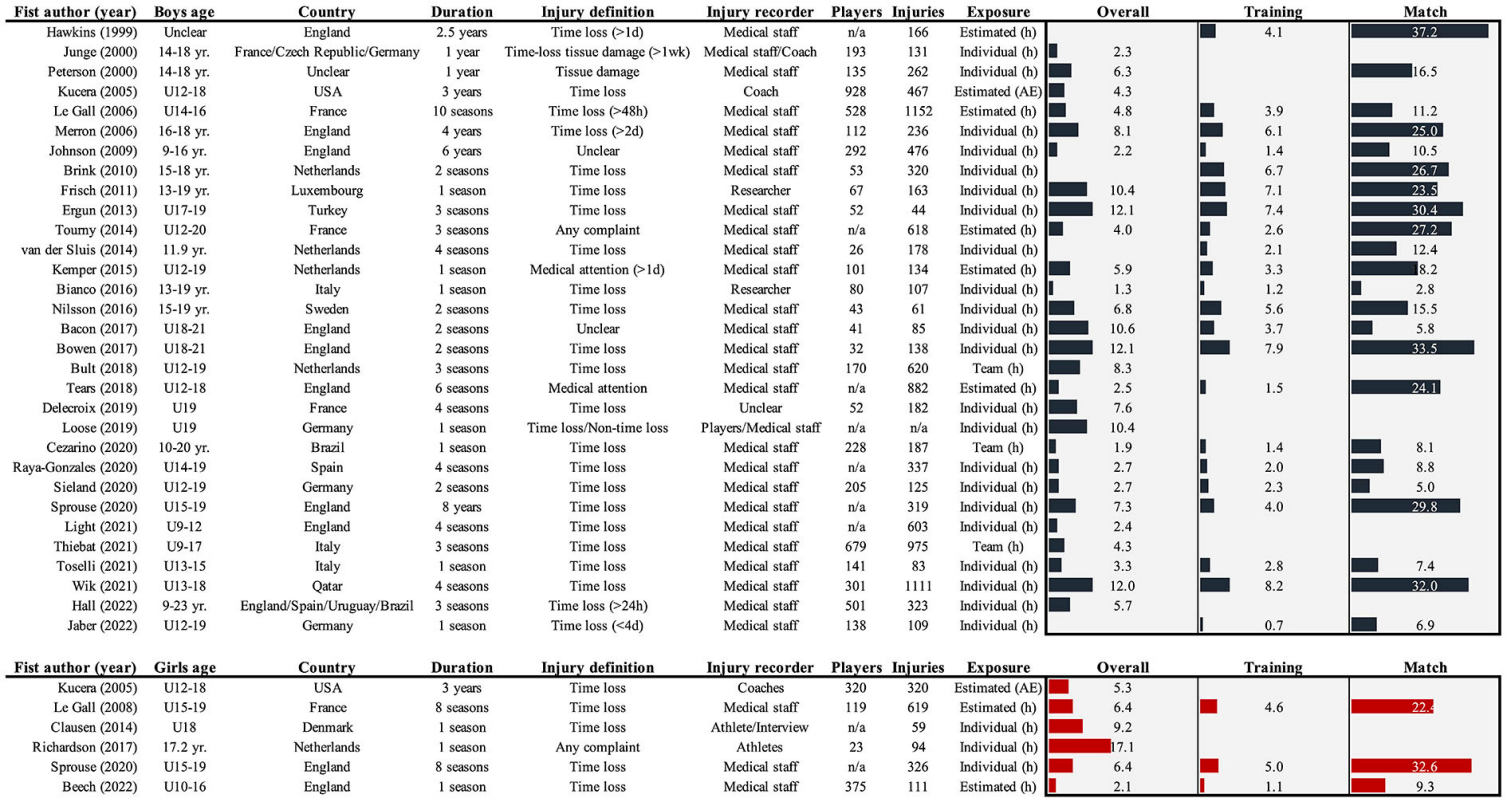
Overview of studies reporting overall, training and/or match injury rates (per 1,000 h or athletic exposures: AE) in high-level youth football players. Where estimates were only provided for subgroups, the average was used to give an idea of the extent, and where rates were presented using multiple definitions, only the narrowest was included (e.g., time loss > medical attention) as these are more comparable between contexts (45). If datasets were overlapping or used in multiple studies, only the main epidemiological study was included.

Counting injuries or calculating the proportion of injured players does not take the time they play football into consideration. This is important, as a team training eight times per week inevitably will see more injuries than a team training once a week; this does not mean that the risk of playing an hour of football is different. Expressing injuries relative to training and match hours is therefore recommended. Using the median of point estimates for reported injury rates (this does not consider the size of each study, nor the uncertainty in their estimates) and keeping methodological differences in mind, the number of injuries per 1,000 h appears similar between genders (Figure 1): around 6.3 (range: 1.3–12.1) for boys (18–20, 24, 26, 27, 29, 31–33, 36, 37, 41, 42, 46–57) and 6.4 (2.1–9.1) for girls (44, 55, 58–60). Matches are consistently associated with greater risk compared to training sessions in both boys (match: 13.4, training: 4.0) and girls (match: 22.4, training: 4.6) (18–20, 24–26, 29, 31–33, 36, 42, 46–50, 53–55, 58, 60–63).

Injury severity is commonly calculated as the number of days elapsed from the day of injury until the day the player returns to full training and/or is available for match selection (64), often presented as the proportion of injuries falling within defined bins (e.g., percentage of all injuries lasting 7–28 days). Although cut-offs vary slightly between studies and the choice of injury definition affects distributions, the combined findings suggest that 38% (7–74%) of injuries in boys last less than a week, another 38% (16–67%) last between a week and a month, while every fifth injury (21%, 2–37%) lasts more than a month (18, 20, 21, 23, 25, 28, 29, 31–37, 39, 41, 46, 48, 49, 53–56, 61). For girls, a larger proportion of “mild” injuries is observed, with around a half (51%, 38–52%) lasting a week or less, a third (36%, 32–41%) between a week and a month, and the remaining 16% (12–20%) more than a month (55, 58, 60).

### What are the most common injury locations and types?

**Figure 2:**
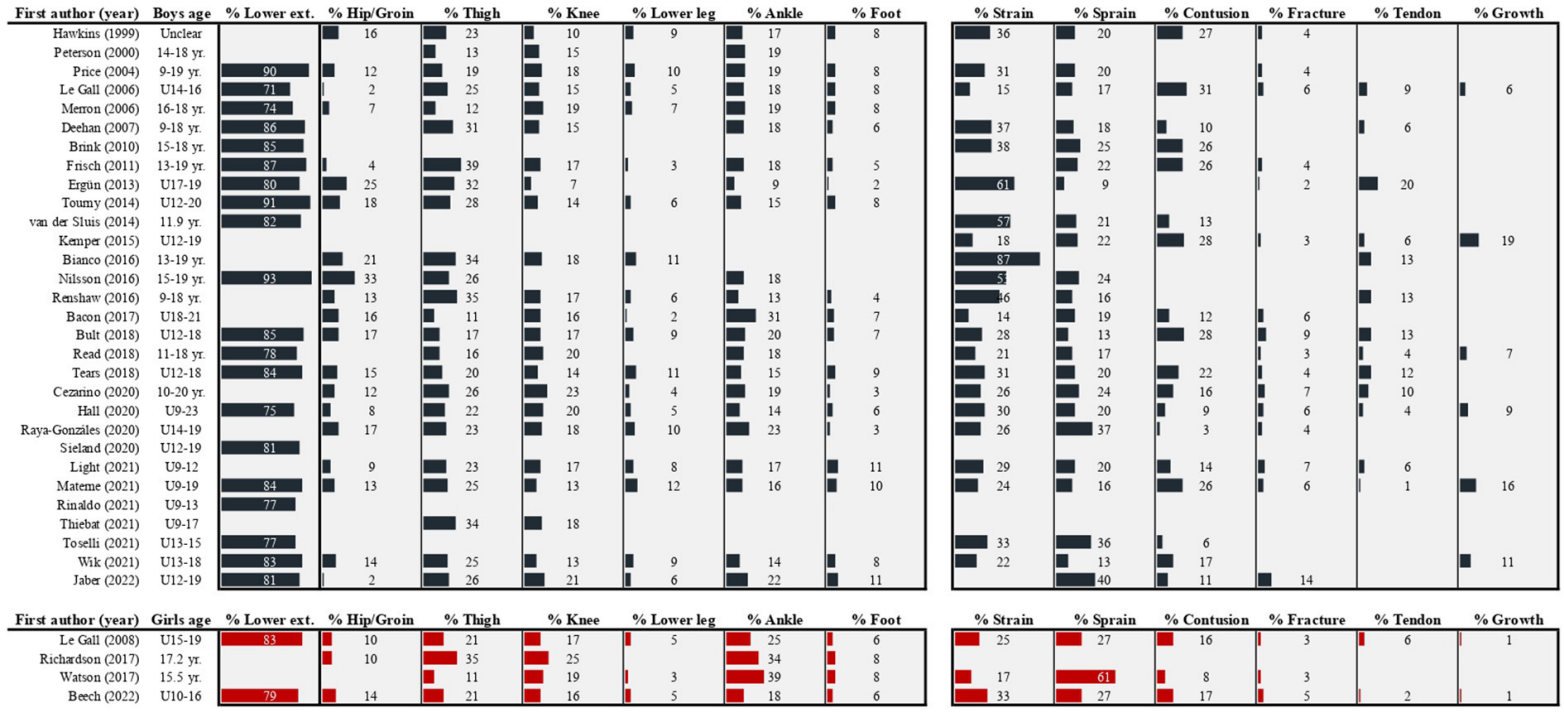
Reported injury proportions within location and type categories in high-level youth football players. Not all studies used the same classifications, and a best effort was made to place injuries in the most applicable category. In studies reporting subgroup proportions without values for the full sample, the average was entered to give an idea of the overall pattern. Where multiple studies were published with the same or overlapping dataset, only the main epidemiological paper was included.

Understanding the injury problem in general is an important first step; however, we need to know *which injuries* are the most troublesome to design impactful injury reduction programmes that target specific mechanisms and risk factors. Given the high running demands and frequent kicking and tackling actions observed in youth football (65, 66), it is perhaps not surprising that the lower extremities are the most affected – accounting for approximately four out of five injuries in boys and girls (Figure 2). Breaking it down to specific body parts, thigh injuries are the most common among boys (median of percentages reported in studies: 25% of all injuries, range: 11–39%), with the ankle (18%; 9–31%), knee (17%; 7–23%) and hip/groin (14%; 2–33%) also common. In girls, ankle injuries are the most common (30%; 18–39%), followed by injuries to the thigh (21%; 11–35%), knee (18%; 16–25%) and hip/groin (10%; 10–14%). Three main injury types can be identified, with strains (31%; 14–87%), sprains (20%; 9–40%) and contusions (17%; 3–31%) together accounting for two out of three injuries in boys. Sprains appear more common among girls (27%; 27–61%), although they together with strains (25%; 17–33%) and contusions (16%; 8–17%) also account for roughly two thirds of all injuries reported. Taken together, these patterns are similar to senior players (67, 68), and it could be argued that football players – for the most part – sustain “typical football injuries” regardless of age and gender when proportions are used.

Isolated proportions for body parts and types are of limited value since they do not consider injury severity, nor do they tell us which injury types to focus on within each location or where different types are located. Reporting injury burden (i.e., days lost relative to hours of football exposure) for combinations of body parts and injury types (or specific diagnoses) would therefore represent an advancement in our understanding (69). Few studies provide this, but there is evidence to show that it changes our interpretation of surveillance data. As an example, a study of academy boys (24) highlighted that thigh muscle injuries were the most common (16% of all injuries, accounting for 11% of total days lost), but joint sprains to the knee had the greatest impact on player participation (only 3% of injuries, but 18% of days lost). Similarly, contusions, which ranked high in terms of proportions, were of low severity and consequently had a relatively low impact (17% of injuries, but only 5% of days lost). Of particular interest to youth populations, injuries to the skeleton were the most burdensome tissue type in this study (23% of injuries, 34% of days lost), although muscle/tendon injuries were the most common (27% of injuries, 16% of days lost). These insights would be lost if severity was not accounted for.

### Do injuries depend on age, growth and maturity?

A wide span in age groups combined with individual differences in timing and tempo of growth and maturation make “youth football players” a heterogeneous population. Some studies will include players closer to childhood, others closer to the senior level, and within age groups there will be variation at the individual level. This section will examine the potential influence age, growth and maturation can have on injury patterns.

#### Age-related injury patterns

In general, injury rates are not the same across age groups; however, the age-related pattern is not unified based on the available research. Several studies indicate that rates increase with age (24, 25, 28, 31, 34, 35, 41, 54), although others report less clear patterns or bell-shaped relationships peaking around the U15-16 groups (20, 23, 26, 27, 29, 30, 37, 39, 48, 53). Injury severity and burden also appears to be influenced by age, often peaking in the U14-16 age groups (21, 24, 35, 37, 53). While mainly examined in boys, increasing injury incidence and burden with age was also observed in a recent study of girls (60), although contrasted by another showing a lower incidence in U19 players compared to U15 (58). An increased risk with age could potentially be explained by players being stronger, faster and heavier as they grow, mature and accumulate training experience. Furthermore, training sessions and matches may be more intense and carry more significance as competition intensifies. Having sustained a previous injury is also more likely with age, which is a strong risk factor for new injuries (70). Changes associated with the growth spurt (e.g., more fragile growth plates, differences in tissue adaptation, decreased bone-mineral density) (71–74) are often used to explain the higher rate of severe injuries and higher burden observed around the years of expected PHV and PWV. Finally, injury trends differ depending on injury type, with more growth-related injuries observed in younger players and more muscle injuries and joint/ligament sprains in older players (21, 24, 28, 29, 35, 39, 56) – likely influenced by players' absolute maturity status.

#### Absolute maturity and injury risk

Some tissues may be more prone to injuries prior to reaching their mature state, such as an underdeveloped brain that appears more prone to concussions, thicker and more fragile cartilage, and a growing skeleton (4). Especially the latter has received attention in youth sports, as skeletal conditions (e.g., Osgood-Schlatter disease) are common and can cause problems for years (75). Some injuries (e.g., fractures through, or extreme load on, the growth plate) have the potential to disrupt normal growth patterns if not managed adequately (76).

*Absolute maturity* (i.e., how close a player is to the mature state) is an interesting concept in terms of injuries. Several studies have demonstrated a pattern where injuries that involve growth areas are less common in players closer to skeletal maturity or adult height (77–79), for whom muscle, joint and ligament injuries are more prominent (78, 79). This likely reflects which tissues and structures represent the “weak link” in the muscle-tendon-skeletal chain; the skeletal attachment site (apophysis) is relatively weaker in immature players, while muscles, ligaments and tendons yield sooner in players with a mature skeleton (80). As consequence, the same mechanisms may lead to different pathologies depending on maturity. For example, a gradual overload may cause apophysitis in an immature athlete but tendinopathy in a mature athlete, and a sudden force may lead to an avulsion in the immature player but a muscle strain in the mature player (80). This theory also aligns with observations that growth-related injuries appear in a bottom-to-top pattern depending on maturity status and age (79, 81), matching the typical distal-to-proximal skeletal maturation process (1, 82).

#### Relative maturity and injury risk

*Relative maturity* (i.e., players maturing earlier or later than others; early, average or late maturer) is perhaps the most obvious concern when discussing maturation and injuries, as this comes back to the issue of early and late maturing players training and competing within the same age groups. Intuitively, the later maturing player would seem more injury prone; however, this is not clear in the literature. Early football studies measuring skeletal age found no differences in overall injury risk between relative maturity categories (i.e., early, on time, late) (47, 78), while two recent studies found that early maturing players actually had a greater risk of injury (77, 83). Studies using anthropometric equations also provide conflicting results, with a Dutch study reporting increased overuse injury risk in later maturing players (but only before and during the year around PHV) (84), an English study reporting no difference in non-contact injury risk between early, normal or late maturing players when PHV-period (pre-, circa-, post-PHV) was accounted for (85), and a Spanish study finding a greater burden of overall and growth-related injuries in late vs. normal maturing players (but not compared to early maturing) before PHV, and no differences between relative maturity categories during or after PHV (40).

#### Rapid growth and injury risk

Although it is difficult to differentiate effects of growth and maturity, higher growth rates (e.g., changes in height, leg length or body mass index) (19, 38, 86, 87) and the circa-PHV period (the months or years around the estimated or observed PHV) (37, 40, 63, 85) have been associated with increased injury risk in high-level youth football. Most studies use relatively broad injury outcomes (e.g., all injuries combined or all overuse injuries), but there is some evidence suggesting that effects of rapid growth are type-dependent, with injury rates for skeletal growth areas particularly elevated during PHV (40). This fits well with the proposed underlying mechanisms for a growth-injury relationship. First, growth plates tend to be thicker and more fragile when growth is at its fastest, making them more susceptible to injuries (71). Second, slower adaptation of tendons and apophyses to a lengthening skeleton compared to muscles may cause increased tension on weaker structures (72). Changes in limb length and mass also increase the force required to move them, which theoretically leads to greater strain on the apophyses (72). Third, delayed bone mineralisation has been observed during rapid growth, coinciding with increased fracture rates; this suggests a period of relative bone fragility (73, 74). Finally, changes in body proportions have been associated with temporarily decreased neuromuscular control (“adolescent awkwardness”), which again may be an injury risk factor (13, 88). While these theories are plausible explanations for players being particularly vulnerable during the adolescent growth spurt, they are rarely included in injury studies and the actual importance of each factor remains unclear.

## Summary

It is clear that injuries are common in high-level male and female youth football players, with strains and sprains to the lower limb dominating among both genders. Injury patterns and the type of injuries recorded do, however, depend on the age group observed, players' absolute maturity status and where a player is in relation to the adolescent growth spurt. This warrants age- and maturity-specific prevention programmes, and one can neither assume that all youth players are the same, nor that interventions that work in senior players automatically transfer to age group football.

### What are the research gaps?

While our understanding of youth injuries is constantly improving, some areas are still lacking. First, our knowledge originates from a relatively narrow sample. Nine out of ten publications at this playing level report injury data only for boys, and there is little to no data on growth or maturation as risk factors in high-level girls' football. Additionally, epidemiology studies are mainly conducted in European settings (85%). Consequently, there is a demographic and geographic imbalance in the literature, mainly considering data from European boys. Second, there is a need for studies with a larger number of injuries to better understand where injury reduction efforts should be focused. There is now sufficient data to confidently say something about injury proportions for separate body parts and injury types (especially in boys), and future studies should attempt to report these in combination, preferably using burden metrics and differentiating match and training injuries. Third, many studies do not record exposure at the individual level. This is essential for accurate estimates of injury incidence and burden, and a requirement to address risk factors such as growth and maturation.

### How can this be used in practice?

Given the pattern of diverse injury locations and types, general prevention programmes (e.g., FIFA 11+ which has been shown to reduce injury risk in young footballers by a third) (89) targeting a large range of potential injuries can be considered appropriate. Keeping the main concepts intact, these can be tailored to suit your specific context (e.g., available time and resources) and increase the chances of successful implementation (90). Detecting and taking pain seriously at an early stage seems important to allow for appropriate management and may limit the time away from sports (91). This may also allow players to continue taking part in sessions, modifying their participation and activities rather than completely removing them from the team (92).

During the adolescent growth spurt and prior to skeletal maturity, it may be necessary to focus more on general movement skills and progressive physical development, being extra careful with increases in load (especially high-impact tasks, such as jumping, acceleration, deceleration) and allowing for sufficient rest and nutrition between sessions (93, 94). Overall load management (e.g., coordinating school, club and regional/national commitments) is particularly challenging, but important, at the youth level (95, 96). Finally, each player must be considered differently. With variations in growth and maturity timing and tempo, individuals will face challenges at different ages. Structured growth and maturity assessments are an option where resources allow it (93, 97); however, observing, talking to, and educating players and parents can perhaps be equally effective. Ultimately, we are all working toward a similar target: to provide players with opportunities to reach their own goals, hopefully enjoying the journey along the way!

## Author contributions

The author confirms being the sole contributor of this work and has approved it for publication.

## Conflict of interest

The author declares that the research was conducted in the absence of any commercial or financial relationships that could be construed as a potential conflict of interest.

## Publisher's note

All claims expressed in this article are solely those of the authors and do not necessarily represent those of their affiliated organizations, or those of the publisher, the editors and the reviewers. Any product that may be evaluated in this article, or claim that may be made by its manufacturer, is not guaranteed or endorsed by the publisher.
